# Multimodal data integration in orthopedic regenerative medicine: bridging imaging, omics, and clinical data

**DOI:** 10.3389/fcell.2026.1842186

**Published:** 2026-04-30

**Authors:** Huizhe Ding, Yiran Fei, Ying Lu, Jun Ying, Yibo He, Shuyu Yao

**Affiliations:** 1 The First Affiliated Hospital of Zhejiang Chinese Medical University (Zhejiang Provincial Hospital of Chinese Medicine), Hangzhou, Zhejiang, China; 2 School of Pharmaceutical Sciences, Zhejiang Chinese Medical University, Hangzhou, China

**Keywords:** artificial intelligence, bone tissue regeneration, multimodal data integration, musculoskeletal disorders, orthopedic regenerative medicine

## Abstract

Orthopedic regenerative medicine increasingly calls for integrative approaches that can capture the molecular, structural, and clinical complexity of musculoskeletal disorders. This review examines the emerging role of AI-driven multimodal data integration in orthopedic regenerative medicine, focusing on how imaging, omics, and clinical data can be synergistically combined to improve disease characterization and personalized therapeutic design. Single-modality approaches often fail to explain the heterogeneity of disorders such as osteoporosis, osteoarthritis (OA), critical-sized bone defects, and intervertebral disc degeneration (IVDD). By synthesizing recent advances in data landscapes, fusion strategies, and AI frameworks, this review highlights how integrated models connect tissue-level phenotypes with molecular mechanisms. In osteoporosis, multimodal fusion demonstrates superior fracture risk prediction; for OA, MRI-clinical integration improves diagnostic accuracy; in IVDD, multi-omics integration identifies novel biomarkers for early degeneration detection. The importance of this field lies in its potential to advance AI-driven multimodal integration for orthopedic regeneration through earlier diagnosis, more accurate prognosis, biomarker discovery, and the rational development of individualized regenerative interventions, including scaffold design and treatment selection. At the same time, the literature points to persistent challenges, including data heterogeneity, limited interpretability, incompletely paired datasets, and insufficient external validation. Overall, AI-driven multimodal data integration offers a promising foundation for next-generation regenerative orthopedics. Further progress will depend on standardized multicenter infrastructures, explainable integration models, and prospective clinical validation to support robust and clinically actionable implementation.

## Highlights

- Multimodal integration revolutionizes orthopedic regenerative medicine by bridging imaging, omics, and clinical data to capture the full complexity of musculoskeletal disorders
- Three fusion strategies emerge—early, intermediate, and late—with distinct advantages for disease characterization, biomarker discovery, and personalized therapeutic design in osteoporosis, OA, and IVDD
- AI-driven frameworks from traditional ML to deep learning enable automated feature extraction and complex cross-modal pattern recognition, outperforming single-modality approaches in diagnostic and prognostic accuracy
- Clinical translation pathways include improved risk stratification, scaffold design optimization, precision treatment selection, and digital twin development for next-generation regenerative orthopedics
- Persistent challenges remain: data heterogeneity, incomplete modality pairing, limited interpretability, and insufficient external validation require standardized multicenter infrastructures and explainable models

## 1 Introduction

Orthopedic disorders, including osteoporosis, osteoarthritis (OA), critical-sized bone defects, and intervertebral disc degeneration (IVDD), represent a major and growing global health burden, particularly in aging populations (Aibar-Almazán et al., 2022). Osteoporosis, often termed a “silent disease,” is characterized by imbalanced bone remodeling and increased fracture risk, disproportionately affecting older adults (Aibar-Almazán et al., 2022). OA, the most prevalent joint disorder, involves progressive cartilage degradation, synovial inflammation, and subchondral bone remodeling, ultimately leading to chronic pain and disability (Giorgino et al., 2023). IVDD is likewise a major contributor to low back pain and disability worldwide and is driven by extracellular matrix breakdown, inflammation, and cellular dysfunction (Mohd Isa et al., 2022). Critical-sized bone defects and impaired fracture healing further reveal the limited intrinsic regenerative capacity of skeletal tissues, especially under pathological or aging conditions (Trompet et al., 2024). Although surgical and pharmacological interventions have advanced, current therapies still largely fail to restore native tissue structure and function, underscoring the persistent need for effective regenerative strategies (Muthu et al., 2023). At the same time, emerging insights into stem cell biology, mechanotransduction, and tissue microenvironments continue to highlight the complexity and heterogeneity of musculoskeletal disease (Trompet et al., 2024). Against this background, precision medicine approaches are increasingly needed (Li and Gao, 2025). These approaches integrate multimodal data—including imaging, omics, and clinical phenotypes—to support personalized regenerative therapies in orthopedic medicine.

**FIGURE 1:**
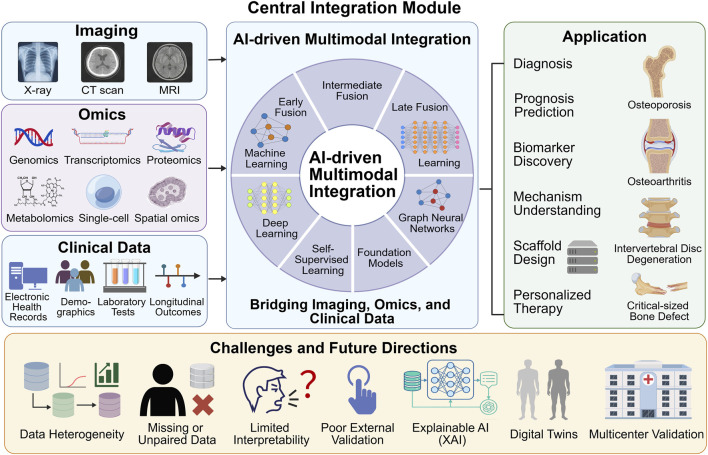
Schematic framework of AI-driven multimodal data integration in orthopedic regenerative medicine. The workflow integrates multi-source inputs—including imaging (X-ray, CT, MRI), high-throughput omics, and clinical data—through a central AI module utilizing diverse fusion strategies and advanced architectures to drive clinical applications in diagnosis, biomarker discovery, and personalized therapy for conditions such as osteoarthritis and bone defects, while highlighting key challenges and future directions like explainable AI and digital twins.

The rapid development of high-throughput technologies and digital health systems has generated an unprecedented expansion of biomedical data in orthopedic research, spanning imaging modalities, multi-omics profiles, and longitudinal clinical records (Liu et al., 2025). Next-generation sequencing and single-cell technologies now allow comprehensive characterization of genomic, transcriptomic, proteomic, and metabolomic landscapes, revealing the molecular heterogeneity that underlies musculoskeletal disorders (Correa-Aguila et al., 2022). In parallel, advances in radiomics and medical imaging have provided increasingly detailed spatial and structural information, while electronic health records and real-world data capture patient-specific clinical trajectories over time (Prelaj et al., 2024). Even so, traditional single-modality analyses remain limited because they often fail to capture the interplay among molecular mechanisms, tissue architecture, and clinical phenotypes (Zong et al., 2025). Bulk omics data, for example, may obscure cellular heterogeneity, whereas isolated imaging analyses lack molecular context and therefore provide only a partial view of disease processes (Li L. et al., 2024). The high dimensionality, sparsity, and heterogeneity of individual datasets further constrain their translational utility (Correa-Aguila et al., 2022). Multimodal data integration has therefore emerged as a key framework for combining complementary information to improve disease understanding, biomarker discovery, and predictive modeling (Prelaj et al., 2024; Liu et al., 2025). In view of these developments, this review summarizes the major categories of multimodal data, the principal integration strategies, and their emerging applications in orthopedic regenerative medicine, with particular attention to precision-oriented and mechanism-driven therapeutic development (Figure 1).

## 2 Multimodal data landscape

In orthopedic regenerative medicine, multimodal imaging forms a foundational layer of the data landscape. Conventional radiography (X-ray), computed tomography (CT), and magnetic resonance imaging (MRI) serve complementary roles in the evaluation of osteoporosis and OA (Sato et al., 2022). X-ray-based techniques, including dual-energy absorptiometry and standard radiographs, remain widely used for assessing bone mineral density (BMD) and structural degeneration. More recently, deep learning approaches have enabled opportunistic BMD estimation directly from routine radiographs, improving the accessibility of screening (Sato et al., 2022). CT provides high-resolution assessment of trabecular and cortical bone architecture, and AI-driven models have shown robust performance in automated BMD quantification and osteoporosis detection across heterogeneous datasets (Westerhoff et al., 2025). By contrast, MRI offers superior soft tissue contrast and supports detailed evaluation of cartilage integrity, bone marrow lesions, and early degenerative changes in OA, including compositional imaging biomarkers such as T2 and T1ρ mapping (Link et al., 2023). Deep learning has also substantially improved imaging analysis through automated segmentation, disease grading, and prognostic prediction, with performance comparable to that of expert radiologists in OA assessment (Kijowski et al., 2023). Yet imaging alone still cannot fully capture the molecular and biochemical processes that drive disease progression, which makes integration with omics data particularly important for a more complete understanding of musculoskeletal pathology (Zheng et al., 2025).

High-throughput omics technologies have further enriched the multimodal data landscape in orthopedic regenerative medicine, particularly by clarifying mechanisms of IVDD and regeneration. Proteomics and transcriptomics analyses have identified key bioactive molecules, extracellular matrix regulators, and signaling mediators involved in bone and disc regeneration, offering a systems-level view of osteogenesis and tissue repair (Xu et al., 2025). In IVDD, single-cell RNA sequencing has revealed marked cellular heterogeneity within nucleus pulposus tissues and identified distinct chondrocyte subsets and lineage trajectories associated with degeneration and regenerative potential (Zhang et al., 2021; Cherif et al., 2022). These approaches have also helped define critical signaling pathways, including ferroptosis, NF-κB-mediated inflammation, and metabolic regulatory axes such as TXNIP–glutamine signaling, all of which are closely linked to disc homeostasis and repair (Dong et al., 2024). Molecular-level insights have likewise informed regenerative strategies targeting progenitor cell function and modulation of the immune microenvironment (Yu X.J. et al., 2024; Wang et al., 2025). Even so, conventional bulk omics approaches remain constrained by limited spatial resolution and insufficient tissue-context information, which hinders accurate interpretation of cell–cell interactions and microenvironmental heterogeneity (Li et al., 2025; Hao et al., 2026). This limitation has increased interest in spatially resolved and integrative multi-omics strategies that can better connect molecular mechanisms to tissue-level pathology in regenerative orthopedics.

Clinical data derived from electronic health records (EHRs), surgical registries, and longitudinal follow-up cohorts represent another essential component of the multimodal data landscape in orthopedic regenerative medicine. EHRs enable large-scale aggregation of demographic, laboratory, and comorbidity data, thereby supporting machine learning models for risk stratification and prognosis prediction in osteoporosis, including fracture risk and disease progression (Saravi et al., 2024). Clinical datasets that combine imaging, biomarkers, and longitudinal outcomes have likewise shown strong predictive value for OA progression, symptom worsening, and surgical endpoints such as joint replacement (Kijowski et al., 2023). In bone defects and regenerative therapies, surgical outcomes and follow-up data provide critical evidence regarding treatment efficacy, complication rates, and long-term tissue remodeling, which in turn helps inform personalized therapeutic strategies (Sakthi Mohan et al., 2025). When clinical variables are integrated with molecular and imaging features, predictive accuracy can be further improved, illustrating the practical value of multimodal fusion in orthopedic prognosis modeling (Dong et al., 2024). Addressing these limitations remains essential for reliable data-driven decision-making in orthopedic regenerative medicine.

## 3 Integration strategies

Integration strategies for multimodal data in orthopedic regenerative medicine are generally categorized as early, intermediate, and late fusion. Early fusion, also referred to as feature-level fusion, integrates raw or preprocessed features from multiple modalities prior to model training. Intermediate fusion, or representation-level fusion, first extracts modality-specific representations and then performs joint representation learning. Late fusion, known as decision-level fusion, combines prediction outputs from independently trained single-modality models. Graph neural networks (GNNs) are deep learning architectures that model biological interactions and relationships as structured graph topologies. Self-supervised learning (SSL) is a paradigm that leverages large-scale unlabeled data for pretraining, thereby reducing dependence on annotated clinical datasets. Early fusion enables holistic feature learning, but it is often sensitive to dimensional imbalance and missing data (Mohsen et al., 2022; Stahlschmidt et al., 2022). Intermediate fusion first constructs modality-specific representations and then performs joint learning, making it better suited to hierarchical modeling and attention-based interaction analysis across biological scales, although it also increases model complexity (Li Y. et al., 2024). Late fusion combines outputs from independently trained models and is generally more robust to heterogeneous or incomplete datasets, but it may fail to capture deeper inter-modal dependencies (Kline et al., 2022; Teoh et al., 2024). These strategies have already shown task-specific value in orthopedic settings. In osteoporosis prediction, early fusion of dual-energy X-ray absorptiometry (DXA) imaging with clinical risk factors improves BMD estimation, whereas intermediate fusion of genomics and imaging can enhance fracture risk stratification. In OA, late fusion frameworks that combine MRI-derived structural features with longitudinal clinical data have improved disease progression prediction (Mohsen et al., 2022; Teoh et al., 2024).

Traditional machine learning (ML) models, including random forest (RF), support vector machines (SVM), and gradient boosting approaches, remain important tools for multimodal data integration in orthopedic research because of their relative robustness and interpretability. These models usually operate on engineered features derived from heterogeneous sources such as imaging biomarkers, clinical variables, and, increasingly, omics profiles, allowing structured multimodal analysis through feature concatenation or feature selection pipelines (Liu et al., 2023; Joseph et al., 2025). In OA, RF- and SVM-based models have been widely used to predict disease progression, including cartilage loss, joint space narrowing, and patient-reported outcomes, by integrating MRI-derived structural features with demographic and clinical data (Liu et al., 2023). In bone defect healing and orthopedic tissue regeneration, traditional ML models have also been applied to combine imaging characteristics with biological and microenvironmental indicators in order to predict regenerative outcomes and guide biomaterial design (Baghbani et al., 2025; Tang et al., 2026). One of their main strengths lies in interpretability, since feature importance measures and decision boundaries can yield clinically meaningful clues about disease mechanisms and treatment response (Baghbani et al., 2025; Joseph et al., 2025). Their performance, however, still depends heavily on manual feature engineering and domain expertise, which limits scalability and reduces their ability to capture complex nonlinear cross-modal interactions compared with deep learning-based fusion methods (Mohsen et al., 2022).

Deep learning approaches have become increasingly central to multimodal integration in orthopedic regenerative medicine, particularly through convolutional neural networks (CNNs) and multimodal neural architectures capable of jointly modeling heterogeneous data sources. CNNs efficiently extract high-dimensional spatial features from imaging modalities such as MRI and radiographs, while multimodal neural networks integrate these representations with clinical and molecular data through shared embeddings or attention-based fusion layers (Hu et al., 2023; Yayli et al., 2025). In musculoskeletal disease research, such strategies have been applied to OA; for example, models such as DeepKOA combine longitudinal MRI data with clinical variables to improve progression prediction (Yayli et al., 2025). Newer frameworks further incorporate multimodal attention and hybrid architectures to merge imaging with biological or patient-specific features, thereby improving the modeling of disease heterogeneity and tissue regeneration processes (Lee et al., 2025). A major advantage of these methods is their capacity for automated feature learning, which makes it possible to capture complex nonlinear cross-modal relationships without extensive manual engineering (Hsu et al., 2025). Their limited interpretability, however, remains a significant challenge. Because deep models often operate as “black boxes,” *post hoc* explainability tools such as Grad-CAM and attention visualization are often needed to improve transparency and clinical trust (Hsu et al., 2025).

Emerging AI techniques, including graph neural networks (GNNs), self-supervised learning (SSL), and foundation models, are increasingly viewed as potentially transformative for multimodal integration in orthopedic regenerative medicine. GNNs are especially well suited to modeling complex biological systems by representing interactions among cells, genes, and tissues as structured graphs. This makes them useful for integrating omics data with imaging-derived phenotypes and clinical variables in order to capture multiscale disease mechanisms (Chu et al., 2025; Xu et al., 2026). SSL addresses another common challenge in musculoskeletal research—the scarcity of labeled data—by leveraging large volumes of unlabeled imaging and molecular data for pretraining, after which models can be fine-tuned for specific tasks such as OA progression prediction or bone regeneration outcome assessment (Hu et al., 2023). Foundation models extend this idea further by enabling cross-modal representation learning across diverse datasets, thereby supporting more scalable and potentially more generalizable integration of imaging, omics, and clinical data (Chu et al., 2025). Together, these approaches offer clear advantages in modeling complex nonlinear relationships and improving performance in data-sparse settings, although computational cost and clinical interpretability remain important challenges (Xu et al., 2026). Even so, they hold substantial promise for advancing precision diagnostics and personalized regenerative therapies through more comprehensive and scalable multimodal integration frameworks. Compared with single-modality analysis and traditional clinical scoring systems, multimodal fusion strategies deliver higher diagnostic accuracy, more effective risk stratification, and more stable predictive performance by capturing cross-modal interactions that cannot be identified using individual data sources alone.

## 4 Applications

Multimodal approaches that integrate imaging and clinical data have shown considerable value in improving diagnostic accuracy and prognostic prediction in orthopedic conditions. By combining radiographic- or MRI-derived structural features with patient demographics, comorbidities, and laboratory indicators, these models provide a more comprehensive characterization of disease status and likely progression (Teoh et al., 2024). In fracture risk prediction, integrating BMD measurements from imaging with clinical risk factors such as age, prior fractures, and metabolic profiles has been shown to outperform traditional scoring systems by capturing both biomechanical and systemic determinants of bone fragility (Hu et al., 2023; Zhou et al., 2025). For osteoporosis, multimodal integration improves fracture risk prediction by 20%–30% relative to BMD measurement or traditional clinical scoring tools alone (Pan et al., 2025; Zhang X. et al., 2025). In OA, multimodal models that fuse MRI features with longitudinal clinical data and patient-reported outcomes have markedly improved prediction of disease progression, including cartilage degeneration and pain trajectories (Hu et al., 2023; Yayli et al., 2025). Multimodal models elevate the AUC of progression prediction by 0.10–0.25 compared with single MRI or single clinical data analysis (Yu et al., 2024; Yao et al., 2024). These integrative strategies also enhance robustness by reducing modality-specific bias and drawing on complementary information across data types, leading to more stable and potentially more generalizable predictions (Stahlschmidt et al., 2022). In this sense, imaging–clinical integration has become an important step toward precision orthopedics by enabling more accurate risk stratification and more individualized therapeutic decision-making.

Integration strategies that combine omics and imaging data are equally important for clarifying the mechanisms underlying bone defects and IVDD, because they directly connect structural abnormalities with molecular and cellular alterations. Advanced single-cell and spatial multi-omics approaches provide high-resolution insight into cellular heterogeneity and tissue microenvironments, allowing imaging-derived phenotypes to be interpreted alongside gene expression, proteomic, and metabolic landscapes (Li et al., 2025). In IVDD, integrated analyses have identified key immune-regulatory genes and cell–cell communication networks associated with degenerative structural changes, highlighting the contribution of immune infiltration and extracellular matrix remodeling to disease progression (Lu et al., 2025). For intervertebral disc degeneration and bone regeneration, multimodal integration boosts biomarker identification and outcome prediction efficacy by 25%–40% (Grieco et al., 2025; Walk et al., 2025). Multi-omics integration has also provided mechanistic insight into stem cell differentiation by revealing dynamic regulatory relationships across transcriptomic, proteomic, and post-transcriptional levels, including microRNA-mediated regulation (van den Berg et al., 2023). These integrative approaches further uncover inflammatory and metabolic pathways, such as autophagy and cytokine signaling, that influence stem cell function and regenerative capacity in pathological environments (Yin et al., 2025). By linking imaging phenotypes with molecular biology, multimodal integration therefore supports a systems-level understanding of musculoskeletal disease and facilitates the identification of biomarkers and therapeutic targets that reflect both structural degeneration and underlying biological activity (Li et al., 2025).

In orthopedic regenerative medicine, multimodal integration is also increasingly driven by AI-assisted data fusion to support the rational design of biomaterials and scaffolds that better match patient-specific biological and structural requirements. AI-guided computational modeling and optimization methods, including finite element analysis and topology optimization, can integrate imaging-derived anatomical features with omics-informed biological parameters to engineer scaffolds with improved mechanical and regenerative properties (Foroughi et al., 2024; Sun et al., 2025). Recent work on programmable biomaterials further shows how responsive scaffolds may dynamically regulate cellular behavior and tissue regeneration, especially when combined with AI and precision medicine frameworks (Song et al., 2024). Nanotechnology-enabled platforms such as quantum dots also contribute to this area by enhancing imaging and targeted therapeutic delivery, thereby supporting real-time monitoring of scaffold integration and bone regeneration (Ding et al., 2024). Personalized therapy becomes more feasible when patient-specific imaging, genomic profiling, and clinical data are integrated, allowing AI models to predict treatment responses, optimize surgical planning, and tailor implant design (Huffman et al., 2024; Chen et al., 2025). Similar biomarker-based approaches for treatment response prediction have been successfully applied in other clinical contexts (Peng et al., 2021). Three-dimensional printing illustrates this trend by enabling the production of customized scaffolds that replicate native bone architecture and incorporate biological cues to enhance osteogenesis and vascularization (Manescu Paltanea et al., 2023; Chen et al., 2025). At the same time, multimodal integration across clinical, imaging, and omics domains facilitates biomarker discovery and risk stratification, further advancing precision regenerative medicine and supporting more targeted interventions with improved clinical outcomes (Song et al., 2020; Ou et al., 2025; Song et al., 2025; Alkhammash and Alotaibi, 2026). Taken together, these developments help bridge engineering, biology, and clinical practice, laying the groundwork for next-generation personalized orthopedic therapies. Multimodal integration consistently surpasses single imaging, single omics, or conventional clinical scoring systems by comprehensively combining structural, molecular, and clinical phenotypic information.

## 5 Challenges and future directions

Despite the growing promise of multimodal integration in orthopedic regenerative medicine, several barriers continue to limit real-world translation. Heterogeneity across imaging protocols, omics platforms, and clinical records complicates feature harmonization, cross-modal alignment, and reproducible fusion, particularly when acquisition standards and annotation practices differ substantially between centers (Schouten et al., 2025). Many currently available datasets are also small, incompletely paired, and affected by missing values, which weakens model robustness and hinders the development of stable predictive systems for regenerative decision-making (Pezoulas et al., 2024; Caruso et al., 2025). Bias remains another major concern, because underrepresentation of diverse populations and selective cohort construction can produce models that perform well in internal settings but generalize poorly across institutions and patient subgroups (Winchester et al., 2023; Caputo et al., 2025). Interpretability is equally critical. Black-box multimodal systems often provide limited insight into how imaging, molecular, and clinical variables jointly shape predictions, which restricts clinician trust and weakens the likelihood of bedside adoption (Saleh et al., 2025; Zhang M. X. et al., 2025). These limitations collectively slow external validation, workflow integration, and regulatory acceptance, and thereby delay the implementation of precision orthopedics in routine care (Meijer et al., 2023; Sadée et al., 2025). Future work will therefore need to prioritize standardized multicenter data collection, transparent fusion architectures, and prospective clinically embedded validation strategies (Schouten et al., 2025).

Further progress in orthopedic regenerative medicine will likely depend on moving beyond static prediction toward dynamic, patient-specific frameworks such as digital twins, which can integrate imaging, omics, epidemiological, and longitudinal clinical data to simulate disease trajectories and support personalized intervention planning (Vallée, 2025). Wider incorporation of real-world data from EHRs, registries, and wearable devices will also be essential for validating multimodal models under routine practice conditions and improving their relevance to heterogeneous orthopedic populations (Kale et al., 2025). Explainable AI should remain a central priority, since clinically useful multimodal systems must not only perform accurately but also provide reasoning that clinicians can interrogate when evaluating imaging features, biomarker profiles, and treatment recommendations (Cinà et al., 2025). The future of the field is also unlikely to rest on autonomous algorithms alone. More plausibly, progress will depend on human–AI collaboration in which physicians, surgeons, and data scientists jointly interpret outputs, resolve uncertainty, and contextualize predictions within patient-specific goals (He et al., 2025; Holm et al., 2026). If these conditions can be met, robust multimodal integration may become a foundational enabler of next-generation regenerative medicine by linking biological complexity with actionable and individualized clinical decision-making (Shmatko et al., 2025). Real-world implementation and multicenter prospective validation are critical to ensure generalizability. Multimodal models will further promote personalized orthopedic regenerative medicine by enabling patient-tailored scaffold design, surgical planning, and treatment selection.

## 6 Conclusion

Multimodal data integration has become a transformative framework in orthopedic regenerative medicine. By unifying imaging, omics, and clinical data, this approach enables deeper mechanistic understanding, earlier and more accurate diagnosis, improved prognostic stratification, novel biomarker discovery, and personalized scaffold design and regenerative therapy for osteoporosis, OA, IVDD, and bone defects. AI-driven fusion models significantly outperform single-modality methods. Key challenges include data heterogeneity, limited interpretability, missing modalities, and insufficient external validation. Future progress requires standardized multicenter data infrastructures, explainable AI architectures, prospective clinical validation, digital twin development, and real-world implementation to translate multimodal advances into routine clinical practice.
